# *PLCL1 *rs7595412 variation is not associated with hip bone size variation in postmenopausal Danish women

**DOI:** 10.1186/1471-2350-10-145

**Published:** 2009-12-23

**Authors:** Stéphane Cauchi, Inger Byrjalsen, Emmanuelle Durand, Morten A Karsdal, Philippe Froguel

**Affiliations:** 1 CNRS 8090-Institute of Biology, Pasteur Institute, Lille, France; 2 Nordic Bioscience A/S, Herlev Hovedgade 207, DK-2730 Herlev, Denmark; 3 Genomic Medicine, Hammersmith Hospital, Imperial College London, London, UK

## Abstract

**Background:**

Bone size (BS) variation is under strong genetic control and plays an important role in determining bone strength and fracture risk. Recently, a genome-wide association study identified polymorphisms associated with hip BS variation in the *PLCL1 *(phospholipase c-like 1) locus. Carriers of the major A allele of the most significant polymorphism, rs7595412, have around 17% larger hip BS than non-carriers. We therefore hypothesized that this polymorphism may also influence postmenopausal complications.

**Methods:**

The effects of rs7595412 on hip BS, bone mineral density (BMD), vertebral fractures, serum Crosslaps and osteocalcin levels were analyzed in 1,191 postmenopausal Danish women.

**Results:**

This polymorphism had no influence on hip and spine BS as well as on femur and spine BMD. Women carrying at least one copy of the A allele had lower levels of serum osteocalcin as compared with those homozygous for the G allele (p = 0.03) whereas no effect on serum Crosslaps was detected. Furthermore, women homozygous for the A allele were more affected by vertebral fractures than those carrying at least one copy of the G allele (p = 0.04).

**Conclusions:**

In postmenopausal women, our results suggest that the *PLCL1 *rs7595412 polymorphism has no obvious effect on hip BS or BMD but may be nominally associated with increased proportion of vertebral fracture and increased levels of osteocalcin.

## Background

Osteoporosis is as a skeletal disorder characterized by compromised bone strength predisposing a person to an increased risk of fracture [1]. Bone mineral density (BMD) is commonly used to diagnose osteoporosis and to predict individual fracture risk [2-4]. Recent genome-wide studies based on hip BMD identified novel susceptibility genes for osteoporosis [5,6]. Besides BMD, a growing body of evidence suggested that bone size (BS) per se also plays an important role in determining bone strength and fracture risk [7-9]. Women not only lose bone density after menopause but also have an increase in skeletal size as a result of periosteal apposition [10]. Previous studies showed that women with hip fractures have larger hip BS [11-20]. Bone must indeed be flexible since it must be able to absorb energy by deforming, to shorten and widen when compressed, and to lengthen and narrow in tension without cracking [21]. Previous studies have demonstrated that BS variation is under strong genetic control, with heritability greater than 50% [9,22-25]. Moreover, segregation analyses have suggested that at least one major gene for BS variation exists in Caucasians [26-28] and in Chinese [29]. Candidate genes have been reported to influence hip BS [30-32]. However, these studies have presented conflicting data, due in part to small sample size and differences in the genetic background of control and case subjects [33]. Recently, the first genome-wide association study to search for novel genes underlying hip BS variation was conducted [34]. Four SNPs associated with hip BS in the *PLCL1 *locus had or approached genome-wide significance level in women. *PLCL1 *encodes an inositol 1,4,5-trisphosphate (IP3) binding protein that can inhibit IP3 mediated calcium signaling [35], an important pathway that regulates the response of bone cells to mechanical signals [36,37]. The most significant SNP, rs7595412, located in intron 3, achieved a p value of 3.72 × 10^-7^. Carriers of the major A allele have around 5 cm^2 ^or 17% larger hip BS than non-carriers. In the present study, we analyzed the effect of rs7595412 on hip BS and osteoporosis traits in 1,191 postmenopausal Danish women.

## Methods

### Subjects

Clinical characteristics of the 1,191 studied individuals were presented in Table 1. The study population consisted of postmenopausal women, from the Prospective Epidemiological Risk Factor study living in the Aalborg area in Denmark [38]. The subjects were recruited by questionnaire surveys offering a screening examination for assessment of skeletal and cardiovascular status. This subpopulation was selected based on those whose BMD and body composition were measured by the same DXA scanner [39,40]. The population characteristics were not statistically significant from the total PERF population indicating that the results reported herein are generally applicable to elderly Caucasian women 60-85 years old [39]. All participating women signed an approved consent form, and the study was carried out in accordance with the Helsinki Declaration II and the European Standards for Good Clinical Practice. The Ethical Committee of Copenhagen County approved the study protocol.

**Table 1:** Clinical characteristics of the studied postmenopausal Danish women

N	1,191
BMI (kg/m^2^)	26.0 ± 3.6
Age (years)	70 ± 5
Gender	Only women
Hip Bone Size (cm^2^)	36.5 ± 3.6
Spine Bone Size (cm^2^)	58.3 ± 5.3
Bone Mineral Density (g/cm^2^)	
Distal part of the arm	0.36 ± 0.08
Femur Neck	0.67 ± 0.10
Total Femur	0.79 ± 0.12
Total Spine	0.88 ± 0.15
Serum Crosslaps (ng/ml)^#^	0.36 [0.19-0.68]
Serum Osteocalcin (ng/ml)^#^	29.27 [19.52-43.88]
Vertebral fractures (Yes/No/na)	214/937/40
Hip fractures (Yes/No/na)	29/1,158/4

Data are presented as arithmetic mean ± standard deviation or as ^#^geometric mean [± standard deviation range]

### Quantitative trait measures

Women underwent a thorough examination. Height and weight were measured to the closest 0.1 cm and 0.1 kg, respectively, to calculate BMI (weight in kilograms divided by the square of height in meters). BMD at the distal forearm was measured by a DTX200 arm scanner (Osteometer MediTech, Rødovre, Denmark). BMD at the lumbar spine L1-L4, total hip, and femoral neck was measured by a Hologic QDR4500 scanner (software version 9.03D; Hologic, Waltham, MA, USA). The hip BS was measured as total bone area (cm^2^) calculated as the sum of the areas of neck, trochanteric, and intertrochanteric regions as measured by DXA and the spine BS (cm^2^) as the sum of the area of L1-L4. The bone formation marker of osteocalcin was measured by the Elecsys N-MID Osteocalcin assay, and the bone resorption marker of the C-terminal telopeptide of collagen type I by the Elecsys CTx assay (Roche, Basel, Switzerland). For these analyses, serum samples were collected in the morning after an overnight fasting period to exclude diurnal variation and the effect of meal. Lateral X-rays of the thoracic and lumbar spine were obtained using standard X-ray equipment. Vertebral deformities from T4 to L4 were assessed by digital measurements of morphologic changes using the Image Pro Image Analyzer software (version 4.5 for Windows; Media Cybernetics, Silver Spring, MD, USA). The ratio of the anterior and posterior heights of each vertebral body was determined digitally, and a difference of >20% between the anterior and posterior edges was considered as a radiographic vertebral fracture. None of the fractures were caused by a traffic accident. Hip fractures were based on self-reported information from a questionnaire.

### Genotyping

The rs7595412 SNP was genotyped using an AOD (assay on demand) kit (Applied Biosystems). The PCR was performed with a GeneAmp 9700 PCR system. The conditions for the TaqMan reaction were 95°C for 10 s and 40 cycles of 92°C for 15 s, 60°C for 1 min, and 15°C for 5 s. Allelic discrimination was performed through capillary electrophoresis analysis, using an Applied Biosystems 3730xl DNA analyzer and GeneMapper3.7 software. The genotypes were determined with an ABI PRISM 7900 HT sequence detection system. There was a 98% genotyping success rate, and the genotyping error rate was assessed by sequencing 384 control and 384 hyperglycemic participants and by re-genotyping a random 10% sample. No difference was found with the first genotyping results; thus, the genotyping error rate was estimated to be 0%.

### Statistical analysis

Multivariate linear regression models, taking into account age and BMI, were performed for testing the association between rs7595412 and osteoporosis quantitative traits. Osteocalcin and crosslaps levels were log-transformed before analysis to obtain normality and symmetry of variances. All *P *values were two-sided. R statistics (version 2.6.1) software was used for general statistical analysis.

## Results

The clinical characteristics of the 1,191 postmenopausal Danish women are presented in Table 1. We genotyped all of them for the rs7595412 SNP and identified 943 A/A, 229 A/G and 19 G/G subjects (Table 2). The genotypic distribution was in Hardy-Weinberg equilibrium (p = 0.24). The *PLCL1 *polymorphism was associated neither with hip BS nor with spine BS variations (Table 2). Furthermore, a BMD test measuring the mineral density (such as calcium) in the distal part of the arm, femur neck, total femur, total spine was performed (Table 2). No association between the rs7595412 SNP and BMD levels was found. Serum osteocalcin and crosslaps concentrations were also measured in the studied individuals (Table 2). Women carrying at least one copy of the A allele had lower levels of osteocalcin as compared with those homozygous for the G allele (A/A + A/G: 29.18 [19.50-43.65] ng/ml *vs *G/G: 35.57 [21.47-58.93] ng/ml, p = 0.03) whereas no effect on Crosslaps was detected. History of vertebral and hip fractures was also recorded (Table 2). Women homozygous for the A allele were more affected by such fractures than those carrying at least one copy of the G allele (A/A: 18.8% *vs *A/G + G/G: 14.9%, p = 0.04) whereas no effect on hip fractures was observed.

**Table 2:** rs7595412 SNP effects on osteoporosis traits in postmenopausal Danish women

	A/A	A/G	G/G	Log-Additive (p value)	Recessive (p value)	Dominant (p value)
N	943	229	19	na	na	na
Hip Bone Size (cm^2^)	36.5 ± 3.6	36.4 ± 3.7	35.9 ± 4.0	0.26	0.47	0.30
Spine Bone Size (cm^2^)	58.3 ± 5.3	58.1 ± 5.0	57.6 ± 6.2	0.49	0.57	0.55
Bone Mineral Density (g/cm^2^)						
Distal part of the arm	0.358 ± 0.077	0.367 ± 0.072	0.335 ± 0.064	0.45	0.12	0.18
Femur Neck	0.667 ± 0.105	0.666 ± 0.101	0.658 ± 0.075	0.44	0.62	0.47
Total Femur	0.791 ± 0.122	0.784 ± 0.131	0.757 ± 0.083	0.16	0.19	0.24
Total Spine	0.880 ± 0.149	0.890 ± 0.164	0.843 ± 0.122	0.94	0.24	0.66
Serum Crosslaps (ng/ml)^#^	0.359 [0.189-0.684]	0.351 [0.187-0.658]	0.457 [0.230-0.905]	0.68	0.10	0.97
Serum Osteocalcin (ng/ml)^#^	29.19 [19.51-43.67]	29.12 [19.46-43.59]	35.57 [21.47-58.93]	0.36	**0.03**	0.71
Vertebral fractures (Yes/No/na)	177/729/37	34/192/3	3/16/0	na	na	**0.04**
Hip fractures (Yes/No/na)	23/917/3	6/222/1	0/19/0	na	na	0.91

Data are presented as arithmetic mean ± standard deviation or as ^#^geometric mean [± standard deviation range]

p-values are from linear or logistic regression models adjusted for BMI and age

na: a low number of women homozygous for the G allele have had vertebral or hip fractures

## Discussion

Due to its incidence and clinical consequences, osteoporosis followed by vertebral, hip, and forearm fractures represents an outstanding problem of nowadays' health care. Because of its high mortality rate hip fractures are of special interest. The proportion of fractures caused by postmenopausal osteoporosis increases with age. Costs of examinations and treatment of women with postmenopausal osteoporosis and fractures are also increasing and represent a significant amount all over the world. Several risk factors are known in the pathogenesis of osteoporosis, first of all the lack of sufficient calcium and vitamin D intake, age, circumstances known to predispose falling, but also genetic factors. Osteodensitometry by DXA is among the most important method to evaluate osteoporosis, since decrease in BMD, defined as the ratio of the bone mineral content to BS, strongly correlates with fracture incidence. Hip BS, as such, was also found to be a valuable marker for hip fracture [11-20]. Recently, a genome-wide association study identified the rs7595412 SNP (minor allele frequency = 11.7%), located in the *PLCL1 *locus, as strongly associated with hip BS variation in 50-year-old subjects [34].

In the present study of postmenopausal Danish women who were 20 years older, we did not observe such association (minor allele frequency = 11.2%). Women over 65 years of age are at particular risk to develop osteoporosis which may partly explain this lack of association. The age-related changes in bone size after the menopause caused by endocortical resorption and periosteal bone apposition have been shown to occur especially in postmenopausal women with increased bone loss [10] but this point is still under debate [41]. The *PLCL1 *genetic variation was also nominally associated with lower levels of serum osteocalcin. Markers of bone resorption, like Crosslaps, are usually elevated in postmenopausal women with osteoporosis as compared with normal postmenopausal women, but the markers of bone formation, like osteocalcin, are much less elevated and may indeed be decreased [42,43]. This pattern of changes in bone turnover suggests that an extent of imbalance of bone resorption and bone formation occurs in osteoporosis [44]. Serum levels of osteocalcin were previously found to be either lower [45,46], similar [47,48] or even slightly elevated [49,50] in patients with postmenopausal osteoporosis than in the control subjects. In their study, Liu and colleagues did not find any effect of *PLCL1 *SNPs on hip and spine BMD [34]. We confirmed the lack of effect on spine BMD and found no association with femur BMD. However, Liu and colleagues found marginally significant association of the *PLCL1 *SNPs with spine BS [34]. We did not observe any effect on spine BS but our results suggest an effect on the backbone since the proportion of vertebral fractures was slightly higher in postmenopausal women homozygous for the rs7595412 A allele than in the other subjects. At menopause, bone turnover increases, leading to poorer bone quality and if the increased bone resorption is not balanced with bone formation, the risk of fracture increases too [51]. Given the low proportion of hip fractures in our study samples, we could not correctly assess the impact of the *PLCL1 *rs7595412 polymorphism on this phenotype.

Our analyses were hypothesis-driven, based on previous studies, and therefore not adjusted for multiple testing. However, false positive results cannot be excluded, so further studies using a large number of samples are necessary to confirm what was observed on vertebral fractures and serum osteocalcin levels. Furthermore, fine-mapping and functional analyses may help to identify etiologic polymorphisms in the *PLCL1 *gene which may have a higher impact on hip BS and related phenotypes.

## Conclusions

In postmenopausal women, our results suggest that the *PLCL1 *rs7595412 polymorphism has no obvious effect on hip BS and BMD but may be associated with increased proportion of vertebral fracture and increased levels of osteocalcin.

## Abbreviations

BS: Bone Size; BMD: Bone Mineral Density.

## Competing interests

The authors declare that they have no competing interests.

## Authors' contributions

SC managed the study, carried out the genetic analyses and drafted the manuscript. ED carried out the genotyping experiments. IB participated in the design of the study, carried out the genetic analyses and drafted the manuscript. MAK participated in the design of the study and carried out the genetic analyses. PF coordinated the study. All authors read and approved the final manuscript.

## Pre-publication history

The pre-publication history for this paper can be accessed here:

http://www.biomedcentral.com/1471-2350/10/145/prepub
